# Longitudinal positron emission tomography and postmortem analysis reveals widespread neuroinflammation in SARS-CoV-2 infected rhesus macaques

**DOI:** 10.1186/s12974-023-02857-z

**Published:** 2023-07-29

**Authors:** Juliana M. Nieuwland, Erik Nutma, Ingrid H. C. H. M. Philippens, Kinga P. Böszörményi, Edmond J. Remarque, Jaco Bakker, Lisette Meijer, Noor Woerdman, Zahra C. Fagrouch, Babs E. Verstrepen, Jan A. M. Langermans, Ernst J. Verschoor, Albert D. Windhorst, Ronald E. Bontrop, Helga E. de Vries, Marieke A. Stammes, Jinte Middeldorp

**Affiliations:** 1 Department of Neurobiology and Aging, Biomedical Primate Research Centre (BPRC), Lange Kleiweg 161, 2288GJ Rijswijk, The Netherlands; 2 Department of Virology, Biomedical Primate Research Centre (BPRC), Rijswijk, The Netherlands; 3 Department of Radiology, Biomedical Primate Research Centre (BPRC), Rijswijk, The Netherlands; 4 Department of Animal Sciences, Biomedical Primate Research Centre (BPRC), Rijswijk, The Netherlands; 5 Department of Comparative Genetics and Refinement, Biomedical Primate Research Centre (BPRC), Rijswijk, The Netherlands; 6 Department Population Health Sciences, Unit Animals in Science and Society, Faculty of Veterinary Medicine, Utrecht University, Utrecht, The Netherlands; 7 Department of Radiology and Nuclear Medicine, Tracer Center Amsterdam (TCA), Amsterdam UMC, Vrije Universiteit, Amsterdam, The Netherlands; 8 Department of Biology, Theoretical Biology and Bioinformatics, Utrecht University, Utrecht, The Netherlands; 9 Department of Molecular Cell Biology and Immunology, Amsterdam UMC Location Vrije Universiteit Amsterdam, De Boelelaan 1117, Amsterdam, The Netherlands

**Keywords:** COVID-19, Non-human primates, Macaques, PET–CT, TSPO, Neuroinflammation, SARS-CoV-2

## Abstract

**Background:**

Coronavirus disease 2019 (COVID-19) patients initially develop respiratory symptoms, but they may also suffer from neurological symptoms. People with long-lasting effects after acute infections with severe respiratory syndrome coronavirus 2 (SARS-CoV-2), i.e., post-COVID syndrome or long COVID, may experience a variety of neurological manifestations. Although we do not fully understand how SARS-CoV-2 affects the brain, neuroinflammation likely plays a role.

**Methods:**

To investigate neuroinflammatory processes longitudinally after SARS-CoV-2 infection, four experimentally SARS-CoV-2 infected rhesus macaques were monitored for 7 weeks with 18-kDa translocator protein (TSPO) positron emission tomography (PET) using [^18^F]DPA714, together with computed tomography (CT). The baseline scan was compared to weekly PET–CTs obtained post-infection (pi). Brain tissue was collected following euthanasia (50 days pi) to correlate the PET signal with TSPO expression, and glial and endothelial cell markers. Expression of these markers was compared to brain tissue from uninfected animals of comparable age, allowing the examination of the contribution of these cells to the neuroinflammatory response following SARS-CoV-2 infection.

**Results:**

TSPO PET revealed an increased tracer uptake throughout the brain of all infected animals already from the first scan obtained post-infection (day 2), which increased to approximately twofold until day 30 pi. Postmortem immunohistochemical analysis of the hippocampus and pons showed TSPO expression in cells expressing ionized calcium-binding adaptor molecule 1 (IBA1), glial fibrillary acidic protein (GFAP), and collagen IV. In the hippocampus of SARS-CoV-2 infected animals the TSPO^+^ area and number of TSPO^+^ cells were significantly increased compared to control animals. This increase was not cell type specific, since both the number of IBA1^+^TSPO^+^ and GFAP^+^TSPO^+^ cells was increased, as well as the TSPO^+^ area within collagen IV^+^ blood vessels.

**Conclusions:**

This study manifests [^18^F]DPA714 as a powerful radiotracer to visualize SARS-CoV-2 induced neuroinflammation. The increased uptake of [^18^F]DPA714 over time implies an active neuroinflammatory response following SARS-CoV-2 infection. This inflammatory signal coincides with an increased number of TSPO expressing cells, including glial and endothelial cells, suggesting neuroinflammation and vascular dysregulation. These results demonstrate the long-term neuroinflammatory response following a mild SARS-CoV-2 infection, which potentially precedes long-lasting neurological symptoms.

**Supplementary Information:**

The online version contains supplementary material available at 10.1186/s12974-023-02857-z.

## Introduction

The coronavirus disease 2019 (COVID-19) is caused by severe acute respiratory syndrome coronavirus 2 (SARS-CoV-2) and primarily manifests as an infection of the respiratory tract, however other organs, including the brain, are affected as well [1, 2]. While most individuals experience relatively mild symptoms, others may progress to develop acute neurological symptoms such as anosmia, dysgeusia, fatigue, diminished consciousness, or memory deficits [3–8]. Approximately 10% of infected individuals suffer from post-COVID syndrome or ‘long COVID’, and have a long-lasting condition, which often includes neurological and cognitive symptoms [9]. Persistent neurological symptoms, most often manifested by a severe form of fatigue, in long COVID patients significantly impact their quality of life [10, 11].

To date, the underlying mechanisms causing neurological symptoms, both in acute and long COVID patients, are poorly understood. Recent research suggests the role of neuroinflammation in this process as was suggested by positron emission tomography (PET) [12] and the presence of activated glial cells in the brain [13–15]. Neuroinflammation can be visualized by radiolabeled PET tracers that bind to the outer mitochondrial membrane 18-kDa translocator protein (TSPO) [16, 17]. TSPO is universally expressed in all organs of the body [18]. In the brain, increased TSPO PET signal is detected in neuroinflammatory and neurodegenerative diseases such as multiple sclerosis (MS) and Alzheimer’s disease [18–22]. In general, TSPO PET signal in the central nervous system (CNS) localizes mainly to microglia and astrocytes depending on the neuropathological context and progress of the disease [16, 17, 23]. TSPO has also been described in vascular cells within the CNS during health and disease [23–25]. However, recent studies have raised the question whether TSPO binding in PETs depicts a neuroinflammatory response as also homeostatic microglia express TSPO [26, 27]. The TSPO PET signal observed also depends on the study performed with different disease pathologies and animal models used [17, 23]. Future studies on the cellular binding and function of TSPO are needed to elucidate the role of TSPO during neuroinflammation [17, 23].

Postmortem analysis of brains obtained from patients severely affected by COVID-19 revealed astrogliosis and microgliosis in the olfactory bulb, cerebellum, brainstem, and hippocampus [1, 13–15, 28]. Moreover, leakage of the blood–brain barrier (BBB) and vascular pathology was demonstrated as well as endothelial cell activation contributing to the infiltration of monocyte-derived macrophages and T cells [15, 29, 30]. These neuroinflammatory responses can cause neuronal damage and possibly initiate neuropathology [14, 31, 32]. Research on the spatiotemporal process and detailed pathophysiological mechanism of SARS-CoV-2 associated neuroinflammation is needed to understand how acute and persistent neurological symptoms develop.

Different animal models have illustrated the neuroinvasive properties of SARS-CoV-2 and the virus’s potential to cause neuropathology [33–35]. In addition, when analyzing brain tissue, microglia activation and T-cell infiltration is observed, indicating ongoing neuroinflammation [36, 37]. Non-human primates (NHPs) have been proven suitable models to study the longitudinal infection process after exposure to SARS-CoV-2, using PET–CT, and postmortem immunohistochemical analysis [38–40]. NHPs infected with SARS-CoV-2 demonstrate a mild-to-moderate form of lung inflammation [38–40]. This study is the first to use [^18^F]DPA714 PET–CTs to visualize the neuroinflammatory process over time in NHPs following a mild-to-moderate SARS-CoV-2 infection. Furthermore, the cell types which were responsible for the increase in TSPO PET signal observed in the hippocampus and pons, were investigated by analyzing TSPO expression postmortem in astrocytes, microglia and brain endothelial cells of infected and control animals.

## Materials and methods

### Ethics approval

This study was performed at the Biomedical Primate Research Centre (BPRC, Rijswijk, Netherlands) under project license AVD5020020209404 which was issued by the relevant national authority (Central Committee for Animal Experiments) according to Dutch law, article 10a of the “Wet op de Dierproeven”. Further approval was obtained after assessment of the study protocol by the institutional animal welfare body.

All procedures, husbandry, and housing were performed in accordance with the Dutch laws on animal experimentation and the EU Directive 63/2010. The BPRC is accredited by the American Association for Accreditation of Laboratory Animal Care (AAALAC) International.

### Animals and experimental interventions

Four Indian-origin male rhesus monkeys (*Macaca mulatta*) (Table 1) from the breeding colony of the BPRC, Rijswijk, The Netherlands, were included. All animals were naïve and classified healthy according to physical examination and the evaluation of routine hematology and serum chemistry before inclusion in the study.

**Table 1:** Rhesus macaques used in the study

Animal code	Age (years:months)	Sex (male/female)
SARS-CoV-2		
R1	4:7	M
R2	4:5	M
R3	7:6	M
R4	7:7	M
Average	6:1	
Controls		
R5	6:5	M
R6	6:4	M
R7	6:10	F
Average	6:3	

During the entire study, the animals were socially housed in pairs. They were offered a daily diet consisting of commercial monkey pellets (Ssniff, Soest, Germany) supplemented with vegetables and fruit. Homemade and commercially available enrichment products were provided daily. Drinking water was available ad libitum via an automatic watering system. Animals were checked at least twice a day for general behavior and were scored for clinical symptoms. After infection, only minor clinical signs putatively related to SARS-CoV-2 were noticed in the animals, i.e., an occasional cough. No change in weight (kg) of all infected animals was observed during the course of the study (Additional file 1: Fig. S1).

**Fig. 1:**
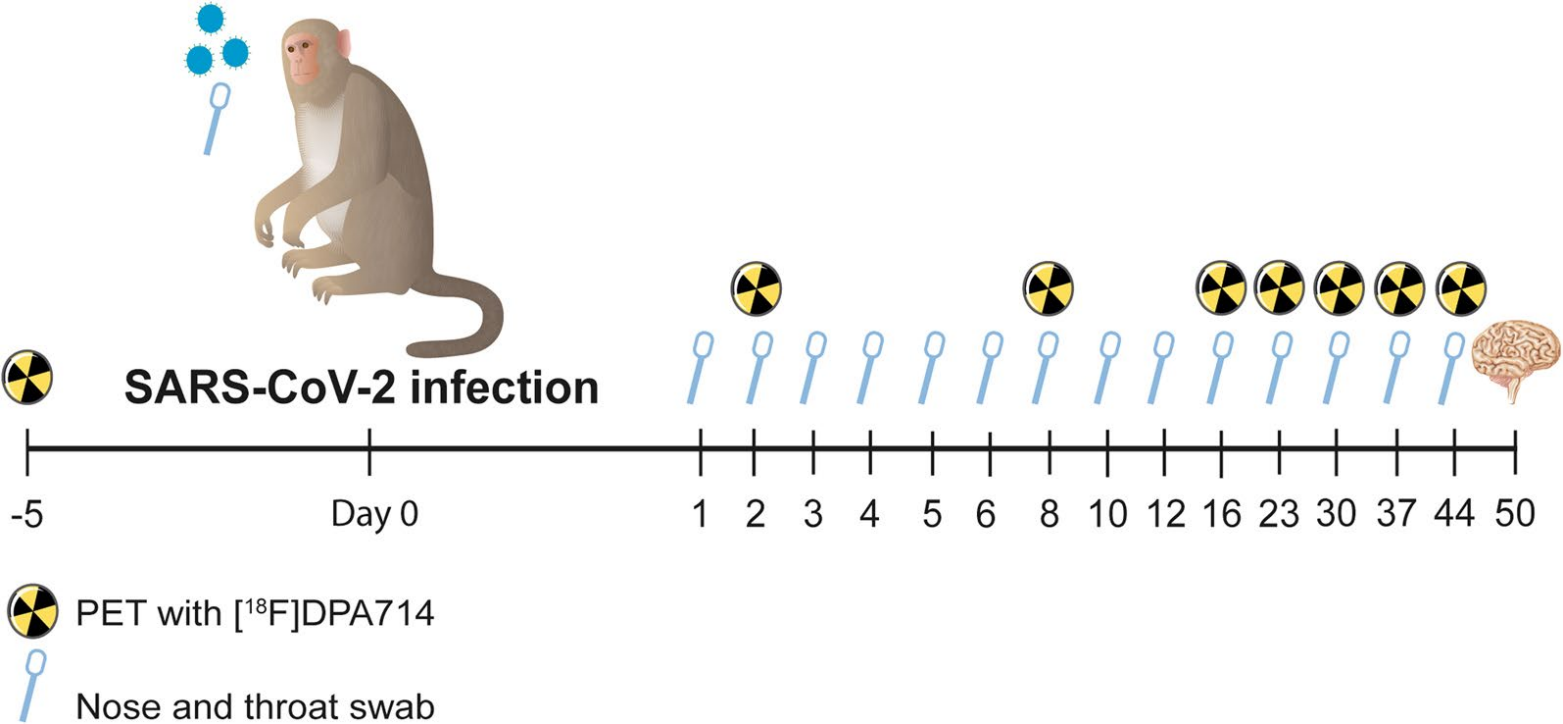
Schematic overview of the experimental set-up of SARS-CoV-2 infection. On day 0 four animals were exposed to SARS-CoV-2. On the day of infection, every day in the following week, and several times after that, nasal and throat swabs were obtained for viral RNA detection. Weekly PET–CTs were performed on all four animals using the ligand [^18^F]DPA714. On day 50 the animals were euthanized, and the brain was collected for further analysis. The right hemisphere was fixed for immunohistochemical analysis, and the left hemisphere was stored for viral RNA detection

All experimental interventions (intratracheal and intranasal infection, nasal and throat swabs and PET–CTs; Fig. 1) were performed under sedation. Animals were fasted overnight and were sedated with ketamine (10 mg/kg ketamine hydrochloride (Alfasan Nederland BV, Woerden, The Netherlands) combined with medetomidine hydrochloride (0.05 mg/kg (Sedastart; AST Farma B.V., Oudewater, The Netherlands), both administrated intramuscularly. After the intervention, upon return of the animals to their cage, atipamezole hydrochloride (Sedastop, ASTFarma B.V., Oudewater, Netherlands, 5 mg/ml, 0.25 mg/kg) was administrated intramuscularly to antagonize medetomidine.

### Virus infection and detection

The animals were infected with SARS-CoV-2, strain hCoV-19/Netherlands/NH-RIVM-27142/2021, the Delta variant (lineage B.1.617.2). The animals were inoculated with a dose of 1 × 10^5^ TCID_50_, diluted in 5 ml phosphate-buffered saline (PBS), via a combined intratracheal, just below the vocal cords (4.5 ml) and intranasal (0.25 ml in each nostril) route. Nasal and throat swabs were taken at regular time points post-infection. The determination of the presence of SARS-CoV-2 messenger RNA and subgenomic messenger RNA in the nasal and throat swabs was performed as described previously using reverse transcription quantitative PCR [39]. Moreover, brain regions were freshly isolated immediately following euthanasia and stored at -80 degrees for reverse transcription quantitative PCR of viral RNA using the same method.

### PET–CT

#### Radiosynthesis of [^18^F]DPA714

Radiosynthesis of [^18^F]DPA714 was performed using procedures described previously [20, 38] using in-house built automatic devices. [^18^F]DPA714 was produced with an average molar activity of 77.4 GBq/μmol (range 39.8–150.6 GBq/μmol at time of injection), a radioactivity concentration of 277.0 ± 172.8 MBq/ml, and a radiochemical purity of at least 98.0%.

#### Scan acquisitions

A baseline PET–CT was acquired pre-infection (day-5) to set a reference parameter. Weekly PET–CTs were obtained (Fig. 1) using a MultiScan Large Field of View Extreme Resolution Research Imager 150 PET–CT (Mediso Medical Imaging Systems Ltd., Budapest, Hungary) as described before [41]. The animals were positioned head-first supine. Following a scout-view, an intravenous bolus (1–2 ml) of approximately 180 MBq [^18^F]DPA714 was administered. All animals underwent the same scan procedure and number of scans, 8 scans total per animal [38]. All scans were obtained on the same day following the same order of animals. The PET image obtained 20–30 min post-injection was used for longitudinal analysis throughout the whole study. Afterwards a CT was acquired to use for attenuation correction. The system utilizes cone-beam CT technology which covers a volume of 150 × 200 × 200 mm^3^ in a single rotation of 32.4 s. For each scan a single rotation of 480 projections was captured. The main scan parameters applied for scans used in this manuscript were 80 kV, 720 μA and an exposure time of 0.09 s. After the scan, upon return of the animals to their cage, atipamezole was administrated intramuscularly to antagonize medetomidine.

#### Reconstruction of PET and CTs 

The emission data were iteratively reconstructed (OSEM3D, 8 iterations and 9 subsets with an isotropic voxel size of 0.8 mm) into a single frame PET image normalized and corrected for attenuation, scatter, and random coincidences using the CT, and corrected for radioactive decay.

#### PET–CT analysis

The analysis was performed with VivoQuant 4.5 (Invicro, Boston, USA). Based on the structures and regions available in the cortical hierarchy atlas of the rhesus monkey (CHARM) [42] and the subcortical hierarchy atlas of the rhesus monkey (SARM) [43] a selection of several regions of interest (ROIs) was made to fuse with the PET–CT data obtained within the study (Additional file 1: Table S1). These ROIs were combined to be able to maintain the general position of the structures towards each other but also to adjust them to the brain of each animal. The fusion and the necessary adjustments are based on both the PET and CT data. Afterwards the standardized uptake values (SUV) of the ROIs were calculated resulting in an average signal of the ROI represented by the SUV_mean_ and an average SUV within a 1-mm^3^ spherical volume around the voxels with the highest value by the SUV_peak_.

### Necropsy and brain tissue sampling

At the end of the study, day 50 pi, animals were anesthetized with a combination of ketamine (10 mg/kg) and medetomidine (0.5 mg/kg) and subsequently euthanized with Euthasol (60 mg/kg) (all intravenous). Brain tissue from the three comparably aged rhesus macaques, which were euthanized for other purposes and stored in the brain biobank (Table 1), was used as control tissue in immunohistological analyses.

After euthanasia, necropsies were performed according to a standard protocol. The brains were separated into two hemispheres as described previously [36]. The right hemisphere was fixed in 10% neutral buffered formalin for 72 h. After fixation, tissues were transferred to 0.1% formalin in PBS, after which the cerebrum, cerebellum and pons were dissected in different coronal parts (0.5 cm) and routinely processed into paraffin blocks. Multiple consecutive 5-μm sections were prepared using a microtome (HistoCore MULTICUT R, Leica) for immunohistochemical staining. From the left hemisphere freshly isolated brain regions were stored at − 80° for qPCR of viral RNA. The brain regions isolated for viral RNA detection: pituitary gland, olfactory bulb, substantia nigra, medulla oblongata, dorsal motor nucleus, pons, brainstem, anterior part of the cerebellum, motor cortex medial, sensory cortex, frontal basal cortex, hippocampus, caudate nucleus, hypothalamus, globus pallidus, putamen, and thalamus.

### Immunofluorescence and quantification

Immunofluorescent staining for TSPO, IBA1, GFAP and collagen IV was performed on tissue sections of the hippocampus and pons of each animal. First, sections were deparaffinized, rehydrated and subsequently quenched with 0.1% w/v glycine in demineralized H_2_O or serum diluted in PBS. For antigen retrieval, slides were steamed in a kitchen steamer for 1 h, while immersed in IHC-Tek epitope retrieval solution (IW-1100, IHC world). After cooling down and washing with PBS, slides were incubated overnight at 4 °C with primary antibodies goat-anti-IBA1 (1:100, MyBioSource, MBS242148), mouse-anti-GFAP (1:200, Sigma-Aldrich, SAB5201104), biotin rabbit-anti-collagen IV (1:200, Abcam, ab6581) and rabbit-anti-TSPO/PBR (1:1000, Abcam, ab109497) diluted in universal antibody dilution buffer (U3510, Sigma-Aldrich). The slides were then washed with PBS and incubated with the secondary antibodies donkey-anti-goat (FITC, IgG, Jackson ImmunoResearch, 705-095-003), donkey-anti-mouse (FITC, IgG, Jackson ImmunoResearch, 715-096-151), streptavidin Alexa Fluor 488 (Invitrogen, S11223) and donkey-anti-rabbit (Alexa Fluor 594, IgG, Jackson ImmunoResearch, 711-585-152) diluted in universal antibody dilution buffer (U3510, Sigma-Aldrich) for 2 h at room temperature. After washing, slides were incubated with Hoechst (1:2500 in PBS) and mounted with prolonged diamond anti-fade mountant or immediately mounted with prolonged diamond anti-fade with DAPI (Invitrogen).

Fluorescent images were acquired using a whole slide scanner microscope (Olympus VS200). Images were collected from the hippocampus and pons. ImageJ (Fiji, Java) and QuPath (version 0.3.2) software were used for picture analyses. Three slides per group were scanned. Three images of the whole hippocampus (scale of 400 μm) and 15 images of the pons (scale of 50 μm) per animal were randomly obtained and used for analysis. The pixels of the fluorescent staining were analyzed by setting a threshold and measuring the area (%) or the signal intensity as the mean gray value. To investigate the number of TSPO^+^ cells and TSPO^+^ glial cells, the number of TSPO^+^, TSPO^+^IBA1^+^ and TSPO^+^GFAP^+^ cells per mm^2^ were automatically counted using 12 images (20x, scale of 50 μm) of the hippocampus per animal in QuPath. The automatic cell count method in QuPath selects cells based on the presence of a cell nucleus and surrounding marker (IBA1 or GFAP). To investigate TSPO in collagen IV^+^ blood vessels, the area and intensity of TSPO within collagen IV^+^ blood vessels were measured using NIS-Elements software (Nikon) analyzing all the collagen IV^+^ blood vessels within three images per animal per region.

### Statistical analysis

Normality and lognormality were tested using the Shapiro–Wilk test. When normally distributed an unpaired *t*-test with a Welch’s correction was used to compare two groups, otherwise a Mann–Whitney U t-test was applied. To investigate correlations a Spearman correlation was performed. Tests were done in GraphPad Prism 9.0 software, *p*-values ≤ 0.05 were considered significant. Ns was considered no significant difference.

## Results

### Increased TSPO PET signals after SARS-CoV-2 infection of rhesus macaques

**Fig. 2:**
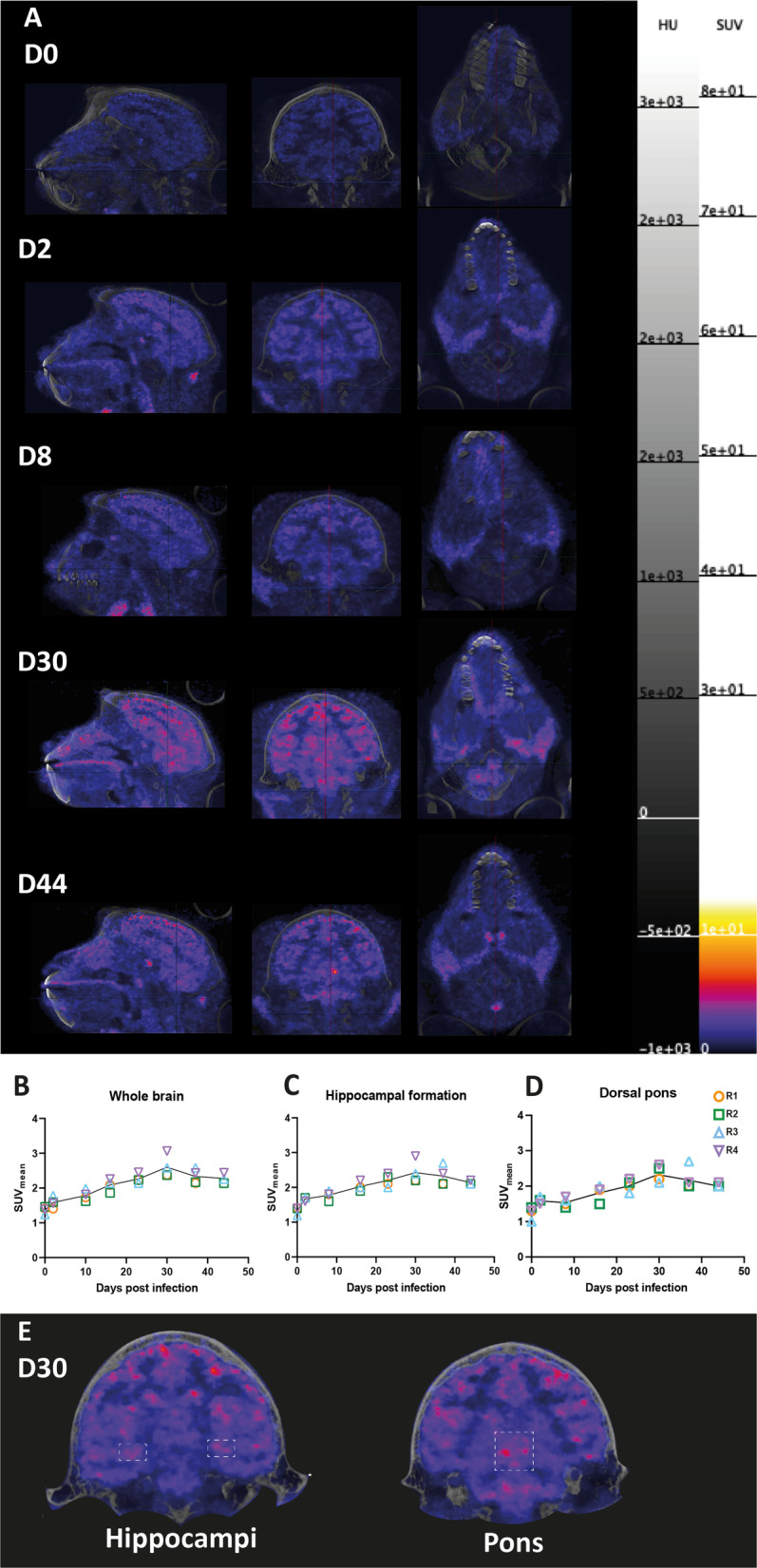
TSPO PET signal increased in the SARS-CoV-2 brain. [^18^F]DPA714 PET–CTs of animal R1 on day 0, 2, 8, 30 and 44 demonstrating an increase in the TSPO signal throughout the brain, including the hippocampus and pons. PET–CTs are demonstrated in a sagittal (left), coronal (middle) and axial (right) manner (**A**). SUV_mean_ of whole brain (**B**; *r* = 0.905, *p* = 0.005), hippocampal formation (**C**; *r* = 0.833, *p* = 0.015) and dorsal pons (**D**; *r* = 0.881, *p* = 0.007) of all four animals during the infection is demonstrated with a peak at day 30. The symbols represent the different animals, and each separate symbol is the SUV_mean_ obtained 20–30 min post-injection of the TSPO tracer ligand. R is Spearman’s correlation coefficient (**B–D**). The hippocampi and pons are demonstrated in a coronal view at day 30 pi. These ROIs are indicated with white dotted boxes to display the increased TSPO PET signal at this timepoint in these brain regions (**E**)

Following exposure with SARS-CoV-2, active replicating virus was indicated at multiple time points up to day 10 pi through detection of subgenomic messenger RNA in the nasal and tracheal swabs (Additional file 1: Table S2) [38]. Although viral RNA was still detected at later time points in the swabs as well (until day 44 pi) (Additional file 1: Table S2), viral RNA was absent in all brain regions at day 50 pi. Despite the absence of viral RNA, we were able to follow SARS-CoV-2 induced neuroinflammation over time with weekly PET–CTs using the radiolabeled tracer [^18^F]DPA714. A radiolabeled ligand needs time to accumulate within a ROI to achieve a stable target to background ratio reflected by the PET signal. Therefore, we used a PET–CT scan obtained 20–30 min post-injection for the in depth analyses of longitudinal data. The scans, indeed, showed a continuous increase in tracer uptake over the course of infection throughout the brain (Fig. 2A; Additional file 1: Fig. S2). The average peak in increase was reached at day 30 pi, with an average SUV_mean_ of 2.6, and afterwards the signal decreased (average SUV_mean_ of 2.3 at day 44) but did not drop to the pre-infection level (SUV_mean_ of 1.4 at day 0) (Fig. 2B). The [^18^F]DPA714 uptake increased approximately twofold (range 1.4–2.0 fold) from day 2 to day 30 pi and demonstrated a significant correlation during the course of 44 days for all four animals (*r* = 0.905, *p* = 0.005) (Fig. 2B). When investigating the four infected animals separately, a similar increase in SUV_mean_ was observed throughout the entire brain (Fig. 2B). To determine the potential contribution of specific brain regions, the SUV_mean_ of ten ROIs of the left and right hemisphere were analyzed. No major differences were observed between regions of different hemispheres and all regions showed an increased signal, which was higher at day 30 or day 37 compared to day 44 (Additional file 1: Table S1). Further analyses were focused on two brain regions, namely the hippocampus and dorsal pons, based on previous results from SARS-CoV-2 infected animals [31, 36, 37, 44]. Both regions displayed a significant increase in tracer uptake (Fig. 2E) and correlation in SUV_mean_ signal during the course of infection in all four animals (hippocampal formation: *r* = 0.833, *p* = 0.015; dorsal pons: *r *= 0.881, *p* = 0.007) (Fig. 2C, D). The four animals individually also demonstrated an increase in SUV_mean_ in these ROIs, with maximum SUV_mean_ values of 2.6 (range 2.2–2.9) for the hippocampal formation (Fig. 2C) and 2.5 (range 2.2–2.7) for the dorsal pons (Fig. 2D). Three of the four animals (R1, R2 and R4) reached this maximum in SUV_mean_ at day 30, however for both the hippocampal formation and dorsal pons R3 showed the highest SUV_mean_ at the next scan obtained at day 37 (Fig. 2C, D). Also, the SUV_peak_ of the whole brain (*r* = 0.810, *p* = 0.022) (Additional file 1: Fig. S3A) and both ROIs (hippocampal formation: *r* = 0.952, *p* = 0.001; dorsal pons: *r* = 0.810, *p* = 0.022) (Additional file 1: Fig. S3B, C) of all four animals showed a significant increase and correlation. An average maximum SUV_peak_ was reached at day 37 in the whole brain at 12.0 (range 10.9–15.1), in the hippocampal formation at 4.7 (range 4.3–5.0) and in the dorsal pons at 3.7 (range 3.3–4.1).

### Increased TSPO expression in hippocampus and pons of SARS-CoV-2 infected animals

**Fig. 3:**
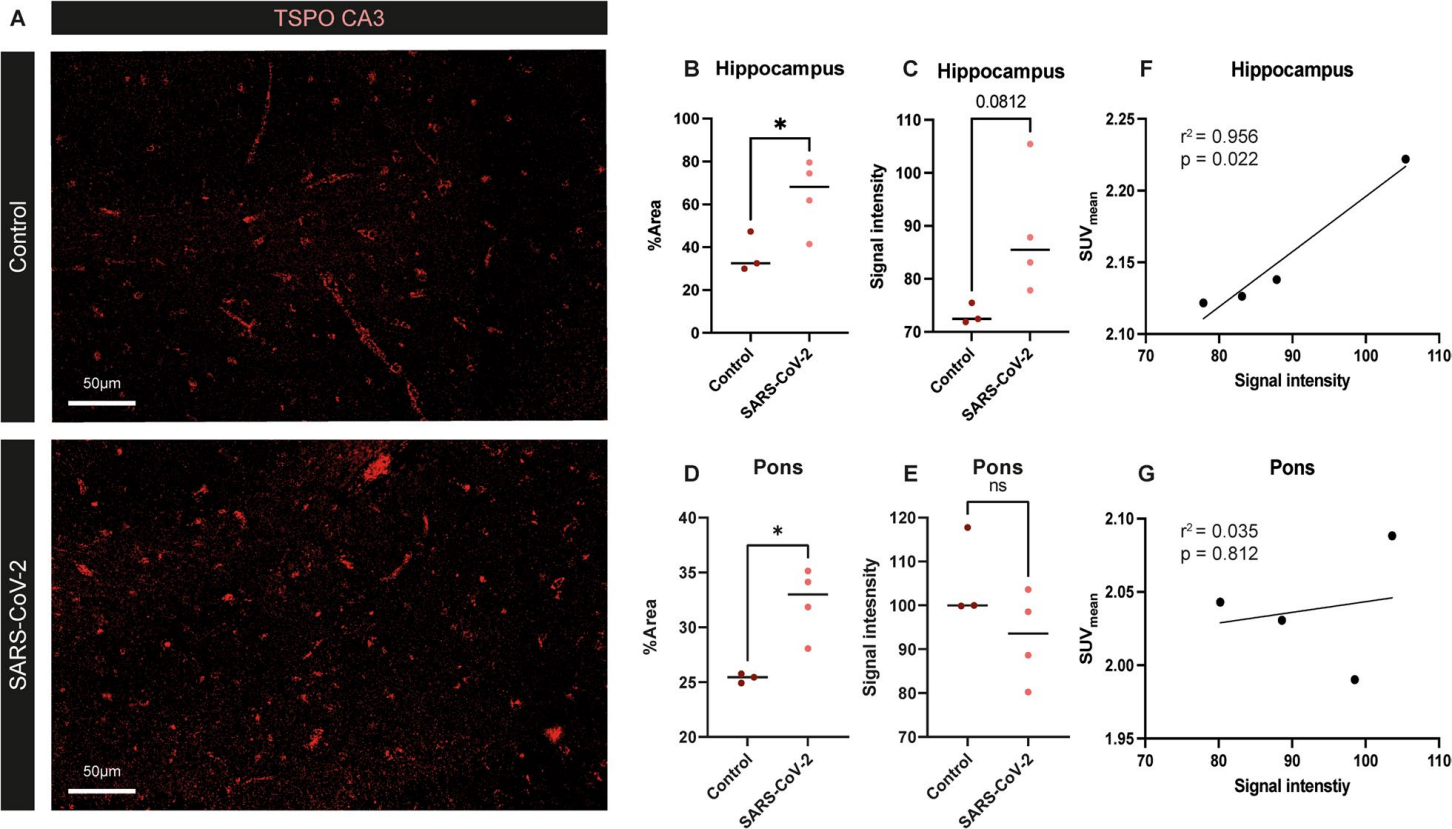
Increased TSPO expression in the hippocampus and pons correlates with TSPO PET signal in SARS-CoV2 infected animals. Representative images of TSPO expression in the hippocampus of SARS-CoV-2 infected macaque (R1) and non-infected control macaque (R5) (**A**). A significant increase in TSPO^+^ area (*p* = 0.042, *t* = 2.757, *df* = 4.762) was observed in the hippocampus of SARS-CoV-2 infected macaques (**B**) and a trend towards an increase (*p* = 0.081, *t* = 2.513, *df* = 3.208) in TSPO signal intensity (**C**). In the pons the TSPO^+^ area was also significantly increased (*p* = 0.020, *t* = 4.354, *df* = 3.144) in SARS-CoV-2 infected macaques (**D**), but the TSPO signal intensity did not differ between the two groups (*p* = 0.229, *U* = 2) (**E**). A significant positive correlation (*r*^2^ = 0.956, *p* = 0.022) was demonstrated in the hippocampus of SARS-CoV-2 infected macaques between the TSPO signal intensity and TSPO SUV_mean_ observed on PET–CTs (**F**) but not in the pons (*r*^2^ = 0.035, *p* = 0.812) (**G**). Representative pictures of the CA3 are used from animals R1 and R5. Results are presented as mean ± SD following Welch’s *t*-test between two groups (**B–D**). Results in **E** are presented as mean ± SD following a Mann–Whitney U *t*-test. For the correlations (**F, G**), *r*^2^ is the simple linear regression coefficient demonstrated. *p < 0.05 means significant difference

To investigate whether the observed increase in TSPO PET signal was accompanied by an increase of TSPO expression, we further analyzed postmortem tissue of the hippocampus and the pons. Immunohistochemical staining for TSPO demonstrated a significantly increased TSPO^+^ area throughout the hippocampus (*p* = 0.042, *t* = 2.757, *df* = 4.762) in SARS-CoV-2 infected animals compared to control animals (Fig. 3A, B). When analyzing the mean signal intensity of TSPO immunoreactivity, the hippocampus showed a trend towards an increase (*p* = 0.081, *t* = 2.513, *df* = 3.208) (Fig. 3C). In the pons, the TSPO^+^ area was also significantly increased in SARS-CoV-2 infected macaques compared to controls (*p* = 0.020, *t* = 4.354, *df* = 3.144) (Fig. 3D). However, no difference was observed in the TSPO signal intensity in the pons (*p* = 0.229, *U* = 2) (Fig. 3E). A significant positive correlation was observed between the postmortem TSPO signal intensity and the in vivo SUV_mean_ at day 44 pi, in the hippocampus (*r*^2^ = 0.956, *p* = 0.022) (Fig. 3F) but not in the pons (*r*^2^ = 0.035, *p* = 0.812) (Fig. 3G).

### TSPO is expressed in microglia, astrocytes and endothelial cells, with most significant increase in astrocytes after SARS-CoV-2 infection

**Fig. 4:**
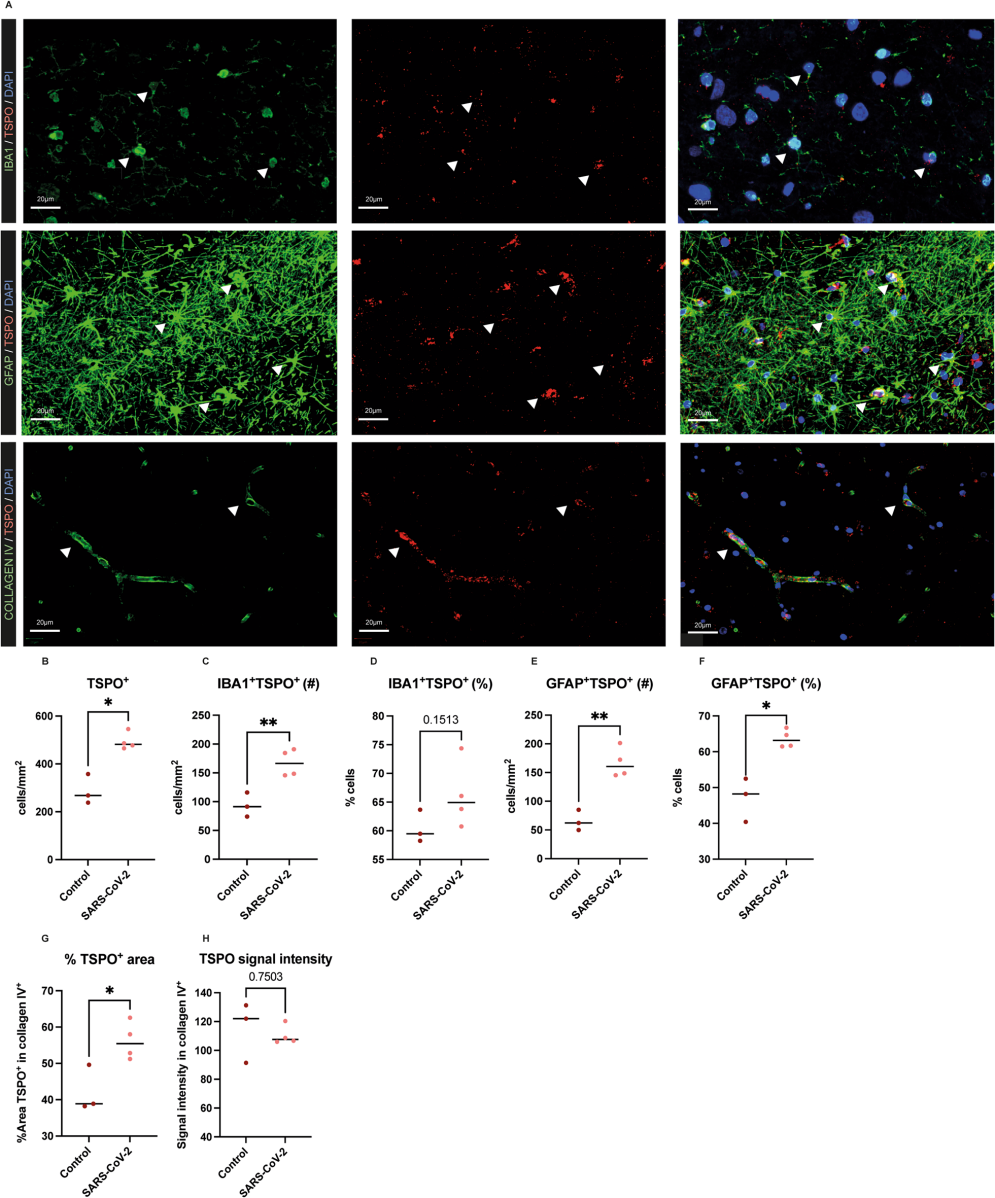
TSPO expression is increased in astrocytes, microglia, and blood vessels in the hippocampus of SARS-CoV-2 infected rhesus macaques. TSPO co-localized with IBA1^+^ and GFAP^+^ glia cells and collagen IV^+^ blood vessels (**A**). Representative pictures are used from animals R1 and R4, and arrowheads point to examples of cells showing co-localization of TSPO in IBA1^+^ and GFAP^+^ cells and in collagen IV^+^ blood vessels (**A**). TSPO^+^ cells were significantly increased in the hippocampus of SARS-CoV-2 infected macaques (*p* = 0.015, *t* = 5.124, *df* = 2.993) (**B**) as well as the number IBA1^+^TSPO^+^ cells (*p* = 0.008, *t* = 4.339, *df* = 4.741) (**C**). However, the percentage (%) of TSPO^+^IBA1^+^ cells within the total number of IBA1^+^ cells did not differ between groups (*p* = 0.151, *t* = 1.725, *df* = 4.521) (**D**). The number of GFAP^+^TSPO^+^ cells were significantly increased in the hippocampus of SARS-CoV-2 infected macaques (*p* = 0.002, *t* = 6.111, *df* = 4.997) (**E**) as well as the % GFAP^+^TSPO^+^ cells within the total amount of GFAP^+^ cells (*p* = 0.031, *t* = 4.437, *df* = 2.514) (**F**). The area (%) of TSPO within collagen IV^+^ blood vessels was significantly increased in the SARS-CoV-2 infected macaques compared to uninfected controls (*p* = 0.039, *t* = 3.087, *df* = 3.827) (**G**). The signal intensity of TSPO in collagen IV^+^ blood vessels did not differ between groups (*p* = 0.750, *t* = 0.358, *df* = 2.314) (**H**). Results are presented as mean ± SD following Welch’s *t*-test between two groups (**B–H**). *p < 0.05, **p < 0.01 means significant difference

Next, we identified which CNS cell types express TSPO in the hippocampus of the rhesus macaque brain by analyzing co-localization of TSPO-immunofluorescence with IBA1 for microglia, GFAP for astrocytes, and collagen IV for the basement membrane surrounding the endothelial cells, and how these cells are affected by SARS-CoV-2 infection. TSPO-immunofluorescence was observed in astrocytes, microglia and endothelial cells in SARS-CoV-2 infected (Fig. 4A) and control animals. The total number of TSPO^+^ cells in the hippocampus was significantly increased in SARS-CoV-2 infected macaques compared to controls (*p* = 0.015, *t* = 5.124, *df* = 2.993) (Fig. 4B). When analyzing specific cell types, we found that the number of IBA1^+^ cells in the hippocampus is significantly increased compared to controls (*p* = 0.007, *t* = 4.520, *df* = 4.917) (Additional file 1: Fig. S4B) as well as the number of IBA1^+^ cells co-expressing TSPO (*p* = 0.008, *t* = 4.339, *df* = 4.741) (Fig. 4C). However, the percentage of IBA1^+^TSPO^+^ cells did not significantly differ compared to controls (*p* = 0.151, *t* = 1.725, *df* = 4.521) (Fig. 4D). The number of GFAP^+^ cells was also significantly increased in the SARS-CoV-2 hippocampus compared to controls (*p* = 0.009, *t* = 4.168, *df* = 4.869) (Additional file 1: Fig. S4C) as well as the number of TSPO^+^GFAP^+^ cells (*p* = 0.002, *t* = 6.111, *df* = 4.997) (Fig. 4E). An increase in GFAP signal was observed in the hippocampus (*p* = 0.046, *t* = 3.051, *df* = 3.483) (Additional file 1: Fig. S5B) and surrounding the blood vessels in the hippocampus (Additional file 1: Fig. S5A), which was even more notable in the pons of the SARS-CoV-2 infected macaques (*p* = 0.002, *t* = 9.036, *df* = 3.363) (Additional file 1: Fig. S5A, C). Contrary to IBA1, the percentage of GFAP^+^TSPO^+^ cells was significantly increased (*p* = 0.031, *t* = 4.437, *df* = 2.514) (Fig. 4F) in the SARS-CoV-2 infected animals. To analyze TSPO expression in the vasculature, collagen IV immunofluorescence was used, however individual cells could not be counted. Therefore, the percentage area (%Area) TSPO/collagen IV and mean signal intensity was analyzed. Overall collagen IV mean signal intensity did not change in the hippocampus of the SARS-CoV-2 infected animals compared to controls (*p* = 0.181, *t* = 1.594, *df* = 4.333) (Additional file 1: Fig. S6A). Nevertheless, in the pons collagen IV mean signal intensity was significantly decreased in SARS-CoV-2 infected animals (*p* = 0.020, *t* = 4.366, *df* = 3.126) (Additional file 1: Fig. S6B). While investigating the percentage area of TSPO within collagen IV^+^ blood vessels in the hippocampus, a significant increase was observed in SARS-CoV-2 infected macaques in comparison to controls (*p* = 0.039, *t* = 3.087, *df* = 3.827) (Fig. 4G). The intensity of TSPO within collagen IV^+^ blood vessels did not significantly differ between infected animals and controls (*p* = 0.750, *t* = 0.358, *df* = 2.314) (Fig. 4H).

## Discussion

In humans, although SARS-CoV-2 initially infects cells of the respiratory tract, multiple organs may be affected such as kidney, liver, heart and brain [45]. In the light of neurological symptoms, which are both present in acute COVID-19 as well as in patients with post-COVID syndrome, there is a need for studies on the effects of SARS-CoV-2 on the brain. Of particular interest is the neuroinflammatory response, which has been observed in people after a mild SARS-CoV-2 infection as well as after severe COVID-19 even while there is no, or minimal, virus detected in the CNS postmortem [13, 46].

Here we utilized a NHP SARS-CoV-2 infection model to measure the neuroinflammatory response after a mild-to-moderate controlled SARS-CoV-2 infection with the delta variant. A previous infection study with the alpha variant of SARS-CoV-2 already showed a clear neuroinflammatory response in all infected macaques, however PET–CT was performed with [^18^F]FDG, and only two animals demonstrated an increased signal in the pituitary gland [36] demonstrating an increased energy metabolism [47]. For the current study, [^18^F]DPA714 was chosen as a PET tracer to visualize the TSPO signal, in order to measure the neuroinflammatory response longitudinally over the course of seven weeks. Although [^18^F]DPA714 is used in humans to visualize neuroinflammation in neurodegenerative diseases, such as MS [24, 48], longitudinal visualization in humans is challenging and has not been performed after SARS-CoV-2 infections. To our knowledge, only one study has been performed which obtained  a single PET with [^18^F]DPA714 in humans after COVID-19. They reported an increased [^18^F]DPA714 uptake in the brains of two long COVID patients, one who had a mild initial infection, another with severe COVID-19, both experiencing long-lasting neurological symptoms [12].

In our study, a consecutive augmentation in [^18^F]DPA714 PET signal was observed throughout the brain in all four animals in the first six weeks of infection, suggesting a widespread neuroinflammatory response following a mild infection. We observed a continuous increase in TSPO PET signal in hippocampus and pons up to 30 days pi and therefore focused our postmortem studies on these regions of interest. The hippocampus, is a region which is crucial for memory formation and consolidation [49–51], and dorsal pons, part of the brainstem is associated with consciousness and autonomic nervous system functions [52]. Both regions appear to be affected by SARS-CoV-2 infection in various animal models [31, 36, 37, 44]. Moreover, as the hippocampus and pons both have different neurological functions, we were interested in further investigating these regions postmortem analyzing the glial cell response following infection. Additionally, we observed a widespread increase in TSPO PET signal throughout the entire brain, also in other brain regions than the hippocampus and pons. Therefore, it would be interesting to investigate the glial cell response in other brain regions as well following SARS-CoV-2 infection. For instance, the cerebellum can also be affected by SARS-CoV-2 infection demonstrating neuroinflammation in COVID-19 patients [1, 13–15]. Overall, our study shows the clinical potential and significance of longitudinal scanning to investigate widespread neuroinflammation over time in SARS-CoV-2 models.

The use of the NHP model also provides the opportunity to directly correlate the longitudinally in vivo observations with postmortem tissue analysis. The interpretation of TSPO PET signal in relation to postmortem expression of TSPO in glial cells has been a matter of some debate when investigating neuroinflammation [16, 19, 23]. Although many studies have used TSPO radiolabeled tracers to analyze neuroinflammation in diseases such as MS and linked this with pro-inflammatory astrocytes and microglia, doubts have been raised whether TSPO expressing cells are indeed in an activated state. Recent studies imply that the increase in TSPO PET signal is due to an increase in glial cell density and not activation status [26, 27, 53]. In the SARS-CoV-2 infected macaques an increase in the TSPO^+^ area was observed, but not TSPO intensity, in the hippocampus and pons. This coincided with an observed accumulation in the number of TSPO^+^ cells in these brain regions following SARS-CoV-2 infection explaining the enhanced TSPO PET signal. Both TSPO expressing IBA1^+^ and GFAP^+^ cells accumulated in these brain regions of SARS-CoV-2 infected animals. However, when analyzing microglia and astrocytes separately, the percentage of GFAP^+^TSPO^+^ cells within the total number of GFAP^+^ cells increased whereas the percentage of IBA1^+^TSPO^+^ cells within the total amount of IBA1^+^ cells did not. Yet, IBA1 is not only a marker expressed by microglia in the CNS, but also by infiltrated macrophages [54]. Both IBA1^+^ microglia and macrophages are therefore potentially contributing to the increase in IBA1^+^ cells in the SARS-CoV-2 infected animals. Future analysis of more specific markers for both cell types will allow us to distinguish between cells and which cell type is more prominent in the CNS following infection contributing to the neuroinflammatory response observed.

These results implie that there is an enhanced number of GFAP^+^ astrocytes expressing TSPO in the SARS-CoV-2 infected animals. An ex vivo study exposing primary human cortical tissue slices to SARS-CoV-2 showed that 90% of infected cells expressed GFAP and aquaporin 4, suggesting a dominant infection of astrocytes in comparison to neurons and other glial cells [55]. Moreover, in our study the total number of GFAP^+^ cells was elevated in the hippocampus of SARS-CoV-2 infected macaques. A similar increase was observed in the piriform cortex of SARS-CoV-2 infected macaques seven days pi [56]. It remains to be investigated what causes this accumulation of GFAP^+^ cells, since mature astrocytes do not often migrate or proliferate [57, 58]. Overall, these results are in line with the idea that an increased TSPO PET signal correlates with an increased number of glial cells. The enhanced TSPO PET signal that was observed in SARS-CoV-2 infected animals, during the course of infection, implies that the glial cell density is affected by the virus, and that the brain generates a lasting innate immune response. This accumulation in glial cell density following SARS-CoV-2 infection suggests a neuroinflammatory response. Yet, the activation status of the glial cells and morphology of IBA1^+^ cells and if these cells potentially skew towards a more inflammatory ameboid phenotype due to SARS-CoV-2 infection needs to be further investigated.

Besides glial cells, we also observed an increase of TSPO^+^ area within collagen IV^+^ endothelial cells in the hippocampus and pons of SARS-CoV-2 infected animals, which can also contribute to the increased TSPO PET signal. TSPO expression in endothelial cells [24, 25] and enhanced vascular TSPO ligand binding [59] has been depicted in diseases such as MS, Alzheimer’s disease and cerebral small vessel disease, also demonstrating neuroinflammation and vascular dysfunction [24, 25, 59]. In COVID-19 patients dysregulation of the vasculature has been demonstrated showing a decrease in tight junction adhesion proteins, vascular leakage as well as astrocyte and endothelial cell activation potentially contributing to neuroinflammation [15, 29, 32, 60]. In SARS-CoV-2 infected macaques 7 days pi abnormal astrocyte–vascular coupling was observed, suggesting dysregulation of the BBB [56]. In our study, enhanced perivascular GFAP was demonstrated in the SARS-CoV-2 infected animals indicating an activation of astrocytes and astrocyte endfeet around blood vessels. Both the increase of TSPO in blood vessels and perivascular GFAP could point to disruption of the BBB following SARS-CoV-2 infection, also witnessed in COVID-19 patients [29].

Although this study was limited by only  using the delta variant of SARS-CoV-2, a previous NHP study using the alpha variant also showed clear signs of neuroinflammation [36]. The neuroinflammatory response to the more recent omicron variant in NHPs is to date not known. Two recent studies in humans compared post-COVID symptoms following infection with delta and omicron variants, and although one study observed a higher incidence in delta cases [61], the other showed that cases infected with omicron were at comparable risk of post-COVID symptoms to those with delta [62]. It is clear that more research is needed to investigate the differences between variants, also in relation to neurological effects, for which NHPs are a suitable model.

Overall, the elevated [^18^F]DPA714 signal in our NHP model and the preliminary results in long COVID patients [12] raise concerns on long-term neurological effects following a mild-to-moderate infection and how this inflammatory response progresses. Recent research has demonstrated the first signs of neurodegeneration and decline of neurogenesis in COVID-19 patients and SARS-CoV-2 animal models in certain brain regions [28, 37]. In COVID-19 patients oxidative stress, microhemorrhages, and neuro-axonal biomarkers in cerebrospinal fluid have been observed, all of which indicate or can cause neuronal injury [30, 46, 63–65]. These observations may underlie the neurological manifestations such as fatigue, decline in cognitive function, learning and memory as seen in long COVID patients [3, 7, 8].

In summary, this study demonstrates an increased [^18^F]DPA714 PET signal which corresponds to elevated numbers of glial cells suggesting a neuroinflammatory response to a mild-to-moderate SARS-CoV-2 infection. To study these inflammatory responses we show the value of PET with [^18^F]DPA714 as radiotracer to visualize SARS-CoV-2-associated glial and vascular changes in the brain over time, combined with postmortem examination of CNS cell types when investigating viral neurological implications. However, how long the inflammation continues, and what the long-term consequences are, is still unclear. To enhance the understanding of neurological manifestations following COVID-19, more research is evident investigating the viral effects on neurodegeneration and which brain regions are most vulnerable. This will give us more knowledge on the cause of neurological symptoms during COVID-19 and in long COVID patients.

## Supplementary Information

**Additional file 1: Figure S1**. Weight of the animals during the study. **Figure S2**. [^18^F]DPA714 PET-CTs of SARS-CoV-2 infected macaques demonstrating an increased signalthroughout the whole brain. **Figure S3**. [^18^F]DPA714 SUV_peak_ is increased during the SARS-CoV-2 infection. **Figure S4**. Glial cell numbers increased in thehippocampus of SARS-CoV-2 infected macaques. **Figure S5**. Increased GFAP expression surrounding bloodvessels in the SARS-CoV-2 hippocampus and pons. **Figure S6**. Brain region differences in collagen IV between SARS-CoV-2 infected macaques and uninfected controls. **Table S1.** Selected regions of interest (ROIs) analyzed by PET-CT. **Table S2**. Viral RNA (genome equivalents/ml) loads and subgenomic messenger RNA positive and negative results in nose and throat swabs.

## Data Availability

All data analyzed during this study are included in this published article and its Additional files or otherwise available from the corresponding author on reasonable request.

## Abbreviations

COVID-19: Coronavirus disease 2019
SARS-CoV-2: Severe acute respiratory syndrome coronavirus 2
TSPO: 18-KDa translocator protein
PET: Positron emission tomography
CT: Computed tomography
pi: Post-infection
IBA1: Ionized calcium-binding adaptor molecule 1
GFAP: Glial fibrillary acidic protein
PASC: Post-acute sequelae of SARS-CoV-2
MS: Multiple sclerosis
CNS: Central nervous system
NHP: Non-human primate
ROI: Region of interest
SUV: Standardized uptake value
PBS: Phosphate-buffered saline
IHC: Immunohistochemistry
BBB: Blood–brain barrier
CHARM: Cortical hierarchy atlas of the rhesus monkey
SARM: Subcortical hierarchy atlas of the rhesus monkey
RNA: Ribonucleic acid
