# Supplementary material for: Longitudinal positron emission tomography and postmortem analysis reveals widespread neuroinflammation in SARS-CoV-2 infected rhesus macaques
Source: J Neuroinflammation. 2023 Jul 29;20:179. doi: 10.1186/s12974-023-02857-z (PMC10387202; doi:10.1186/s12974-023-02857-z)
Supplement: Supplementary file 1 — Additional file 1: Figure S1. Weight of the animals during the study. Figure S2. [18F]DPA714 PET-CTs of SARS-CoV-2 infected macaques demonstrating an increased signalthroughout the whole brain. Figure S3. [18F]DPA714 SUVpeak is increased during the SARS-CoV-2 infection. Figure S4. Glial cell numbers increased in thehippocampus of SARS-CoV-2 infected macaques. Figure S5. Increased GFAP expression surrounding bloodvessels in the SARS-CoV-2 hippocampus and pons. Figure S6. Brain region differences in collagen IV between SARS-CoV-2 infected macaques and uninfected controls. Table S1. Selected regions of interest (ROIs) analyzed by PET-CT. Table S2. Viral RNA (genome equivalents/ml) loads and subgenomic messenger RNA positive and negative results in nose and throat swabs. [file 12974_2023_2857_MOESM1_ESM.docx]

Additional file 1

**Figure S1. Weight of the animals during the study.** The weight of the SARS-CoV-2 infected animals (kg) is depicted over time during the study. The animals were weighed on the day of inclusion for the study which is depicted with (*****) for each animal. At the start of the study the animals were weighed daily and at the end weekly until the day of euthanasia. No prominent changes were observed in the weight of all four SARS-CoV-2 infected animals during the study.

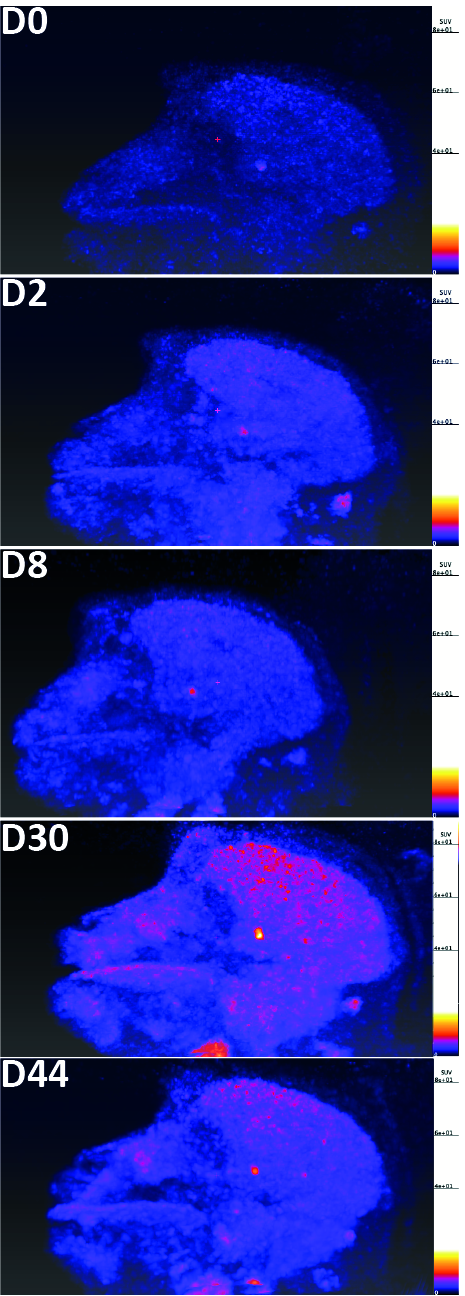


**Figure S2.** **[^18^F]DPA714 PET-CTs of SARS-CoV-2 infected macaques demonstrating an increased signal throughout the whole brain.** Maximum Intensity Projections (MIPs) of the whole brain showing an increase in TSPO signal following SARS-CoV-2 infection.


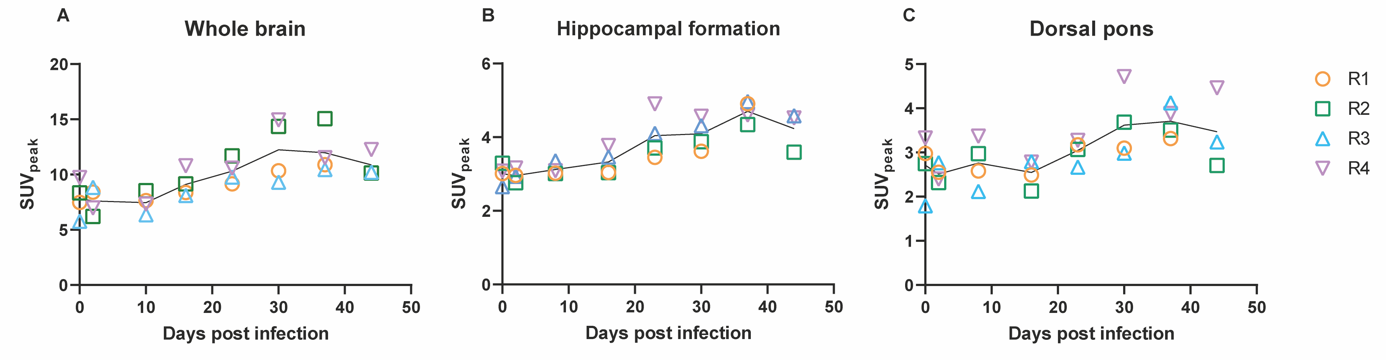


**Figure S3. [^18^F]DPA714 SUV_peak_ is increased during the SARS-CoV-2 infection.** A significant increase is demonstrated of the [^18^F]DPA714 SUV_peak_ in the whole brain (r = 0.810, p = 0.022) (**A**), in the hippocampal formation (**B**; r = 0.952, p = 0.001 and dorsal pons (**C**; r = 0.810, p = 0.022) of all four animals. An average maximum SUV_peak_ is reached at day 37 in the whole brain at 12.0 (range 10.9-15.1), in the hippocampal formation at 4.7 (range 4.3-5.0) and in the dorsal pons at 3.7 (range 3.3-4.1). The symbols represent the different animals, and each separate symbol is the SUV_peak_ obtained 20 minutes post injection of the TSPO tracer ligand. R is Spearman’s correlation coefficient (**A-C**).


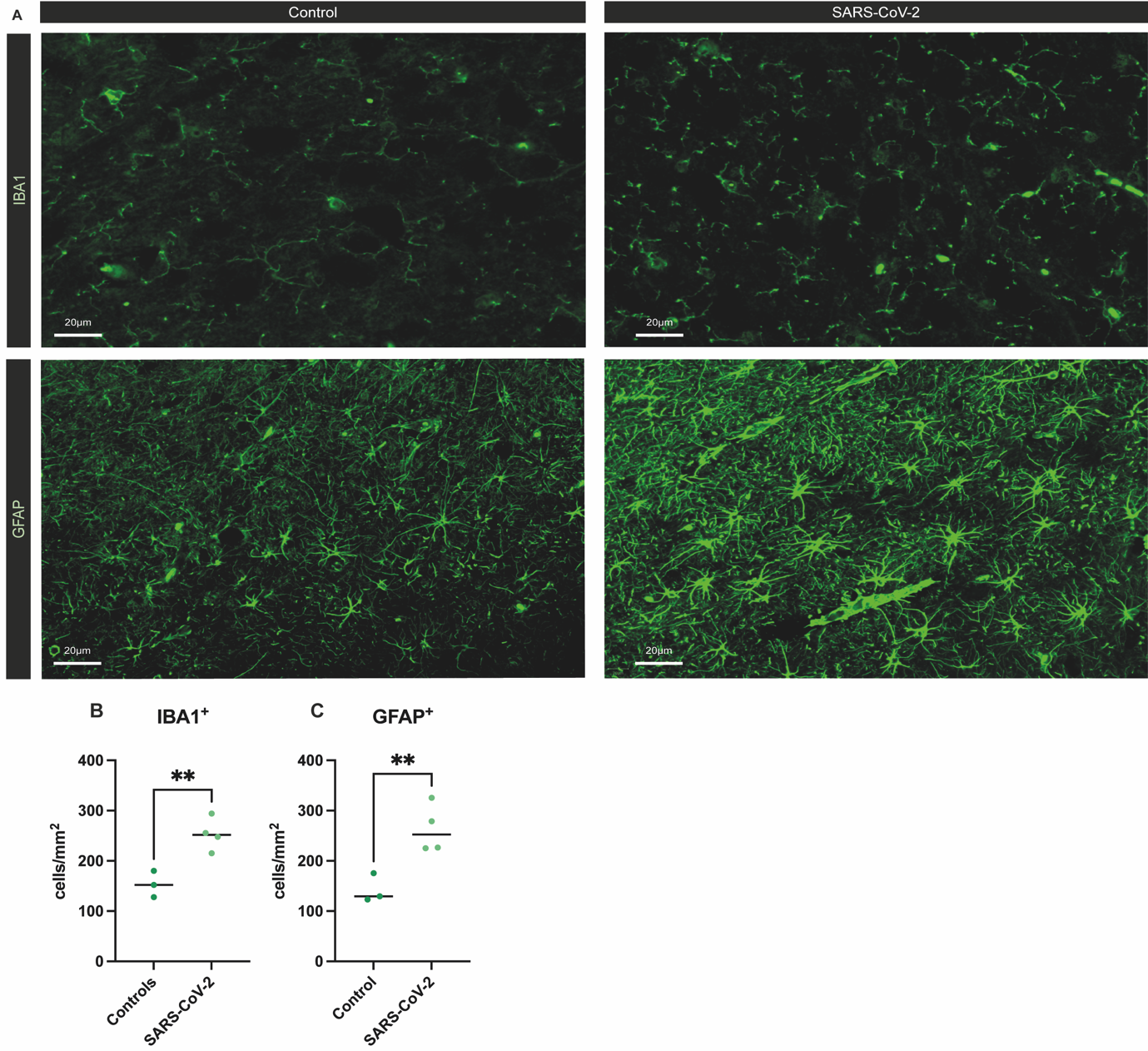


**Figure S4.** **Glial cell numbers increased in the hippocampus of SARS-CoV-2 infected macaques.** Representative pictures of IBA1 and GFAP in control (R5) and SARS-CoV-2 infected macaques (R2) show increased immunofluorescence in SARS-CoV-2 infected macaques (**A**). Quantification shows a significant increase of IBA1^+^ cells (**B**; p = 0.007, t = 4.520, df = 4.917) and GFAP^+^ cells (**C**; p = 0.009, t = 4.168, df = 4.869) in SARS-CoV-2 infected macaques compared to controls. Results are presented as mean ± SD following Welch’s t-test between two groups (**B,C**). **p < 0.01 means significant difference.

**
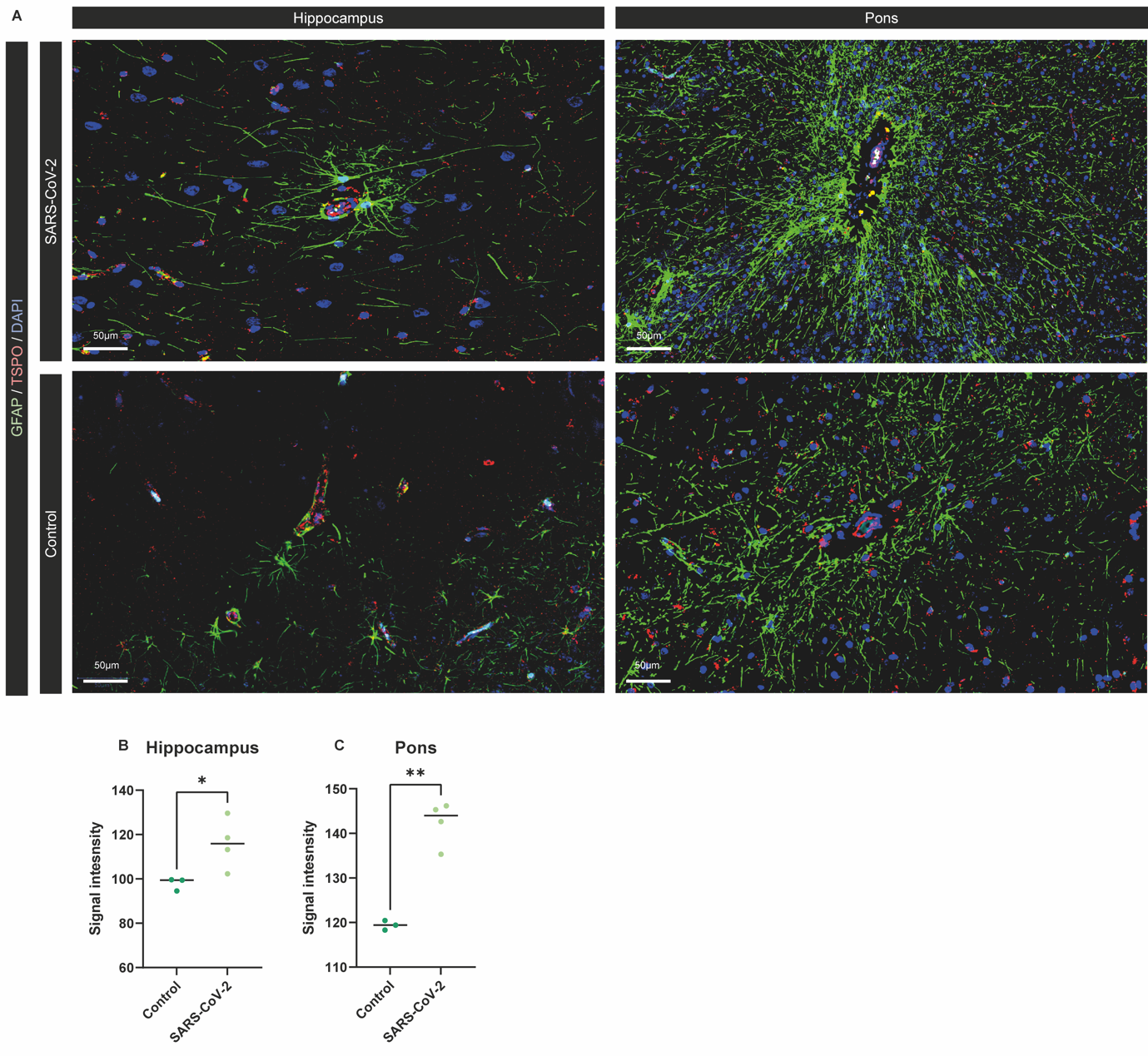
**

**Figure S5.** **Increased GFAP expression surrounding blood vessels in the SARS-CoV-2 hippocampus and pons.** Representative pictures of clear perivascular GFAP expression in the hippocampus of SARS-CoV-2 infected macaques (R2) and to a greater extent in the pons in comparison to controls (R6) (**A**). Quantification shows a significant increase in GFAP expression in the hippocampus (**B**; p = 0.046, t = 3.051, df = 3.483) and pons (**C**; p = 0.002, t = 9.036, df = 3.363) of SARS-CoV-2 infected macaques. Results are presented as mean ± SD following Welch’s t-test between two groups (**B,C**). *p < 0.05, **p < 0.01 means significant difference.


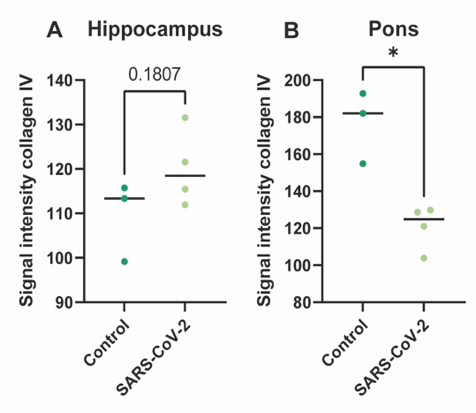


**Figure S6.** **Brain region differences in collagen IV between SARS-CoV-2 infected macaques and uninfected controls.** The signal intensity of collagen IV in the hippocampus did not differ between groups (p = 0.181, t = 1.594, df = 4.333) (**A**). In the pons a significant decrease in collagen IV signal was observed in SARS-CoV-2 infected macaques (**B**; p = 0.020, t = 4.366, df = 3.126). Results are presented as mean ± SD following Welch’s t-test between two groups (**A,B**). *p < 0.05 means significant difference.
